# Editorial: Edible mushrooms and the gut microbiota

**DOI:** 10.3389/fnut.2023.1349429

**Published:** 2023-12-20

**Authors:** Wei Liu, Bei Gao, Fuqiang Yu, Xian Wu, Lingfei Li

**Affiliations:** ^1^Institute of Agro-Food Science and Technology, Shandong Academy of Agricultural Sciences, Jinan, China; ^2^School of Marine Sciences, Nanjing University of Information Science and Technology, Nanjing, China; ^3^The Germplasm Bank of Wild Species, Yunnan Key Laboratory for Fungal Diversity and Green Development, Kunming Institute of Botany, Chinese Academy of Sciences, Kunming, Yunnan, China; ^4^Department of Kinesiology, Nutrition, and Health, Miami University, Oxford, OH, United States; ^5^College of Food Science and Technology, Yunnan Agricultural University, Kunming, China

**Keywords:** edible mushrooms, gut microbiota, disease prevention, polysaccharides, functional foods

Edible mushrooms represent a significant and sustainable food source, notable for their capacity to be cultivated using diverse agro-industrial byproducts and waste materials, circumventing the need for arable land (1). Edible mushrooms have earned recognition as a nutritious “superfood” due to their rich profile of functional substances, including unsaturated fatty acids, bioactive polysaccharides, polyphenols, vitamins, and minerals (2). The consumption of edible mushrooms has been linked to a reduced risk of various diseases, such as obesity, diabetes, digestive ailments, cardiovascular disorders, and several types of cancer (3–7). An increasing body of evidence suggests that the health benefits of edible mushrooms are closely associated with their ability to regulate the gut microbiota (3, 8).

Based on research outcomes from both human populations and animals, particularly with the rapid advancement of multi-omics technologies, it is now well-established that the gut microbiota plays a pivotal role in a variety of physiological functions within the human body (9, 10). This includes but is not limited to, the regulation of nutrient absorption and metabolism, maintenance of energy homeostasis, and orchestration of immune system development and normal function (9, 11). Correspondingly, an imbalance in gut microbiota is intricately linked to an elevated risk of various diseases (12–14). As such, strategies aim at rectifying gut microbiota dysbiosis hold promise for treating and preventing the diseases associated with this perturbation.

The main purpose of this Research Topic is to collect papers to advance the understanding of the interaction between edible mushroom nutrients or bioactive components and the gut microbiota. A comprehension of these scientific intricacies is essential for identifying prebiotics and formulating functional foods derived from edible mushrooms, with the ultimate goal of mitigating and managing gut microbiota-associated disorders.

The bioactive polysaccharides, most notably prebiotics, derived from edible mushrooms are highly noteworthy. Zhao et al. summarized the methods involved in extracting and purifying bioactive polysaccharides from edible mushrooms, as well as the mechanisms by which these edible mushroom-derived polysaccharides (EMPs) promote health by regulating the composition of gut microbiota. The prebiotic effect of EMPs is based on the fact that the human body lacks enzymes to break down complex polysaccharides, rendering them unable to be directly utilized by the digestive tract and primarily degraded by symbiotic gut bacteria (11). Therefore, as a bacterial “fuel,” EMPs can effectively stimulate the proliferation of polysaccharide-degrading bacteria, which are beneficial in most cases (15). The microbiota-modulating function of EMPs is also evident in their ability to effectively ameliorate microbiota dysbiosis associated with diseases and to some extent, intervene in the disease progression (8). Inflammatory bowel disease (IBD) is a highly typical disease closely associated with gut microbiota dysbiosis (16). Sun et al. reported that polysaccharides derived from *Grifola frondosa* (Dicks.) Gray and *Inonotus obliquus* (Fr.) Pilat can alleviate disease symptoms by reversing gut microbiota dysbiosis in a dextran sulfate sodium (DSS)-induced colitis mouse model. In addition to IBD, existing clinical or experimental evidence also suggests that a range of diseases can benefit from the gut microbiota improvement function of EMPs, such as diabetes, liver disease, colon cancer, and Alzheimer's disease (3, 5, 17, 18).

In relation to their gut-microbiota modulating effects, edible mushrooms serve an additional role in boosting the immune system. In a pilot clinical study conducted by Nishimoto et al., three commonly consumed mushroom types in Japan, *Pleurotus eryngii, Grifola frondose*, and *Hypsizygus marmoreus*, were investigated. The study revealed a significant elevation in intestinal short-chain fatty acids (SCFAs) and IgA levels in participants after mushroom consumption. The intestinal IgA is crucial for maintaining the immune barrier of the intestine by neutralizing toxins and viruses, preventing excessive live bacterial adherence or translocation, clearing unwanted macromolecular structures at the epithelial surface, and facilitating the directed sampling of luminal antigens (19). The absence of IgA can directly lead to impaired initiation of the intestinal immune system (19, 20). Xie et al. conducted a study using a mouse model with cyclophosphamide-induced immune dysfunctions, revealing the immune enhancement activity of *Ganoderma lucidum* polysaccharide peptide and its association with gut microbiota-modulating characteristics. Additionally, findings from Nishimoto et al.'s research showed a significant increase in intestinal IgA levels in individuals with higher baseline SCFAs before dietary intervention. This observation implies a close correlation between an individual's baseline gut microenvironment and the outcomes of dietary intervention strategies (21). It underscores the importance of considering individual variations in the microbiota when contemplating the application of dietary intervention strategies for disease prevention or treatment.

In summary, this Research Topic provides new insights into the influence of edible mushrooms on the gut microbiota, elucidating the regulatory effect of this interaction on immune function in both human and animal models and its implications for disease prevention. Despite the considerable amount of research in this area, the extensive diversity among edible mushroom species, the intricate composition of their nutrients and bioactive compounds, and the complex nature of the gut microbiota suggest that investigations into the interactions between edible mushrooms and the gut microbiota are far from exhaustive. Furthermore, as findings from cellular or animal models may not fully mirror their impacts on the human body, it remains essential to conduct additional human intervention trials to comprehensively understand the effects of edible mushrooms and their bioactive compounds on human gut microbiota, as well as the associated health benefits stemming from these effects.

## Author contributions

WL: Writing—original draft, Writing—review & editing. BG: Writing—review & editing. FY: Writing—review & editing. XW: Writing—review & editing. LL: Writing—original draft, Writing—review & editing.
